# Brachial Neuritis and Similar Conditions Involving the Arms

**Published:** 1907-11-23

**Authors:** T. D. Savill


### BRACHIAL NEURITIS AND SIMILAR CONDITIONS.

By T. D. SAVILL, M.D.Lond.

Notes of a Lecture delivered at the West End Hospital for Diseases of the Nervous System.

The causes of neuritis affecting the arms are classified into local and constitutional. The former include wounds, bruises, muscular strains, etc.; pachymeningitis; bone disease; and other spinal affections. Many of these local lesions are found in the most unexpected quarters, and may at first show themselves by no other symptoms than those of the resulting neuritis. For instance, here is a baby with a flaccid paralysis of the right upper extremity, involving especially the deltoid and upper arm muscles. In this case the probable cause is an old fracture of the clavicle, though it is still uncertain exactly where the fracture was, and the attendant practitioner had no suspicion of any such accident having occurred. At any rate, the paralysis is due to some bony pressure on the brachial plexus, and the case is really a surgical one.

Another instructive case is this one, that of a man who came here in June last with extensor paralysis of the right upper extremity. The triceps is all right, but the supinator longus is paralysed, and extensor power in the forearm is lost. This is a case of complete musculo-spiral paralysis due to a lesion of the upper arm. He broke his humerus in a railway accident twenty-three years ago, from which his symptoms date. Neuritis due to injury is, as a rule, a one-sided lesion.

The constitutional causes of neuritis which I have myself met with are: alcohol; tobacco; rheumatism; gout; toxic (not local) effects of tuberculosis and syphilis; lead poisoning; influenza, diphtheria, and other specific fevers; gonorrhœa; pyorrhœa alveolaris; and intestinal sepsis. Others which are admitted to give rise to the condition, but of which I have no personal experience, are cancer and leukæmia, both by their general effects.

The symptoms of brachial neuritis are, briefly, pain, weakness, and wasting, in that order of occurrence. Pain is usually neuralgic in character: it may be continuous and prolonged, and it follows the course of the nerve trunks. Sometimes there are painful spots. The very existence of peripheral neuritis was disputed about fifteen years ago, but tenderness along the course of a nerve trunk can hardly be due to anything else, and this carried conviction.

First of all, I want you to look at this woman, who has double brachial neuritis. In her case there is good reason to suppose that the cause is rheumatism, for she has also joint lesions of that nature. The next case is that of a man who had a carbuncle on the neck in June of this year, and following on that got pain in his left shoulder and down his arm. In time weakness, and ultimately wasting, developed, and in four months his forearm has wasted three-quarters of an inch. There is no stiffness, but perfect flaccidity. The lesion is therefore attacking the lower motor neurons; that is, it is somewhere between the anterior horn cells and the ends of the nerves. There is no increase, but on the contrary loss, of the deep reflexes. The alterations of response to electrical stimulation are difficult to determine satisfactorily in brachial neuritis, partly because of the pain caused. In this case tenderness can be traced down the arm and along the courses of the musculo-spiral, median, and ulnar nerves. What has probably happened is that cicatricial tissue consequent upon the healing of the carbuncle has involved the nerve fibres at some part of their course.

Here is a man who came in November of last year complaining of pain and numbness in both arms for five days. He had also had an attack of lead colic. At that date he had complete wrist drop, but can now extend his hands enough to paint a ceiling, which he regards as a good test. In the right hand especially power has not yet completely returned. In this case the supinator longus was never affected, and it is, in fact, exempt in a considerable number of lead palsies. The reason for this is uncertain, but I believe that it is because the nerve to the supinator longus is a short branch from the main trunk of the musculo-spiral, and that the toxic lesions begin at the ends of the nerves and spread upwards, commencing in those whose course is longest. In severe cases the supinator longus does become involved.

This man illustrates the advantage of certain lines of treatment. He has had iodides and mistura alba; and as local treatment, which is merely palliative and restorative, he has had massage and galvanism. The latter has been applied by a double arm bath; one arm is placed in each bath, and the current is slowly reversed so as to run first one way and then the other. Of treatment for pain in brachial neuritis, the best is dry heat; light baths, heat baths, and Turkish baths are all of value. Analgesic drugs, such as phenacetin, are sometimes useful. If no constitutional cause for the condition can be found, put the patient on salicylate as an empirical measure.

The diagnosis of brachial neuritis is often difficult, because many nervous diseases commence by predominating in the arms. This patient, whom I now show you, came to the hospital about three weeks ago with a history of four years' weakness of the arms, followed by wasting of the thenar and hypothenar eminences, and then by wasting of the arms themselves. Two years ago his legs went the same way. In the early stages this might easily have been mistaken for a case of brachial neuritis, though it is true he suffered no pain, nor has he tenderness over the nerves. He is the subject of an atrophic flaccid paralysis with loss of reflexes, due to a lesion of the lower motor neurons; the main lesion is in the cells of the anterior horns, and is that of progressive muscular atrophy. You will see he still has a slight patellar reflex, due to the fact that some of the nerve fibres which control the quadriceps extensor are still unaffected. In infantile paralysis the same thing can sometimes be observed, and for a similar reason. Another feature of this case which you may observe is the gait; in both progressive muscular atrophy and neuritis the lesion predominates in the extensors of the lower segments, and therefore the gait is similar in the two conditions.

Another disease which may be mistaken for neuritis is amyotrophic lateral sclerosis. Here is a patient, aged 50, whose symptoms began in August 1906, when she noticed weakness in the hands and painful stiffness of the left shoulder-joint. As in the previous case, there is wasting of the muscles of the hands, and some degree of the claw-like main-en-griffe. There is stiffness of the muscles and a marked increase of all the deep reflexes. She has, in short, an atrophic paralysis with stiffness, due to lesions of the pyramidal tracts and of the anterior horn cells. Her gait, as you see, is getting very spastic, whereas last January she could walk perfectly well. I think there is no doubt that she has amyotrophic lateral sclerosis, though at one time I thought of syringomyelia; however, the onset of stiffness has cleared up the diagnosis. These cases are always liable to confusion with neuritis.

Other conditions to be distinguished from brachial neuritis are brachial neuralgias occurring in hysterical or neurasthenic subjects. There are no tenderness, no boring pains, no wasting, and no real paralysis.

That is all I have to say about neuritis, but I can show you an instance of an occupation neurosis, which is a kindred condition. Two years before I first saw this patient, in January last, she lost the power to write with her right hand, and at once began to learn with the left. Then she began to drop her paint-brushes, and after that her sewing-needles too. At that time there was a slight hypertrophy of the right supinator longus, but that cannot be detected now. There are six symptoms, any of which may be exhibited in the subjects of occupation neuroses; they are pain, stiffness, hypertrophy, tremor, weakness, and atrophy. In this case some intestinal sepsis may have been a contributing cause. The patient has had arm baths and resistance movements; she made a complete recovery in June, but left off all treatment, and went back to her painting to make up for lost time. In consequence she had to return last week with a renewal of the condition.

The first indication in these cases is to rest the overworked muscles and to re-educate the movement which is at fault. But in nearly all cases you will find some toxæmia as well. I do not know that I can always identify the particular agency at work, but I find in Post Office employees whom I see that salicylate of soda does many of them good, with attention to general hygiene and knocking them off sugar and meat.

